# Importance of early use of tolvaptan in hyponatremic acutely decompensated heart failure patients, a retrospective study

**DOI:** 10.1186/s43044-024-00603-1

**Published:** 2025-01-13

**Authors:** Rarsari Soerarso, Emir Yonas, Silfi Pauline Sirait, Dian Yaniarti Hasanah, Sunu Budhi Raharjo, Bambang Budi Siswanto, Maarten J. Cramer, Pim van der Harst, Marish I. F. J. Oerlemans

**Affiliations:** 1 Department of Cardiology and Vascular Medicine, Faculty of Medicine Universitas Indonesia, National Cardiovascular Center Harapan Kita, Jakarta, Indonesia; 2 Department of Cardiology, Division of Heart & Lungs, University Medical Center Utrecht, University of Utrecht, Utrecht, The Netherlands

**Keywords:** Acute decompensated heart failure, Hyponatremia, Length of stay, Tolvaptan

## Abstract

**Background:**

Hyponatremia is one of the complicating findings in acute decompensated heart failure. Decrease in cardiac output and systemic blood pressure triggers activation of renin–angiotensin–aldosterone system, antidiuretic hormone, and norepinephrine due to the perceived hypovolemia. Fluid-overloaded heart failure patients are commonly treated with loop diuretics, acutely decompensated heart failure patients tend to be less responsive to conventional oral doses of a loop diuretic, while other different diuretics could work in different part of nephron circulation system. In this study, we aim to further examine the role of tolvaptan, a vasopressin receptor antagonist, in the treatment of hyponatremia secondary to acutely decompensated heart failure.

**Results:**

A total of 71 patients with hyponatremia secondary to ADHF were included, and all patients were given tolvaptan. 37 patients were administered tolvaptan early (up until 5 th day of admission). 34 patients received tolvaptan after 5 th day of admission mean administration as 6.86 th day, and median administration was 5 th day. Analysis showed lower length of stay in patients receiving early administration of tolvaptan compared to late administration (8.86 ± 5.06 vs 18.5 ± 9.05 p0.001, respectively). Patients with early initiation of tolvaptan also achieved a larger net increase in sodium levels at discharge compared to admission (6.46 ± 6.69 vs 3.68 ± 4.70 p0.048, respectively).

**Conclusions:**

Early administration of tolvaptan in treating hyponatremia in acutely decompensated heart failure patients is associated with a lower length of hospitalization and a higher increase in serum sodium of patients in hyponatremic ADHF patients.

## Background

Hyponatremia remains a challenge in the management of acute decompensated heart failure. A decrease in cardiac output and systemic blood pressure triggers the activation of RAAS, and renal conservation of sodium due to the perceived hypovolemia [1]. Hyponatremia is a well-established marker of poor prognosis in ADHF. In patients admitted for ADHF, hyponatremia, which is defined as a serum sodium level < 135 mEq/L, is associated with multiple adverse outcomes including worse short and long-term mortality, higher readmission rates, and longer length of hospital stay [2].

Acute heart failure causes millions of hospital admissions each year, with hyponatremia being a common finding in these patients [3]. Hospitalization and management of ADHF account for the largest proportion of the cost of care.

Fluid-overloaded heart failure patients are commonly treated with loop diuretics, unfortunately, due to a decrease in renal perfusion and the intense activation of the RAAS system, acutely decompensated heart failure patients tend to be less responsive to conventional oral doses of a loop diuretic [4]. This phenomenon is commonly referred to as “diuretic resistance” that is caused by multi-organ interplay between the renal and cardiovascular systems [5]. Diuretic resistance is defined as a failure to increase fluid and sodium output sufficiently to relieve volume overload, edema, or congestion despite a full dose of a loop diuretic, which can be further defined as a failure of oral furosemide (160 mg twice daily or equivalent) to increase sodium excretion of 90 mmol over 3 days [6].

The occurrence of diuretic resistance in patients with ADHF during hospitalization is associated with an increased mortality rate, increased length of stay, and consumption of more medical resources [7–9].

Several studies have shown an association between hyponatremia and the increase in morbidity and mortality in patients who are admitted to the hospital due to heart failure. Hyponatremia is also associated with an increase in rehospitalization rates, increased length of stay, increased complications, and the cost of care while hospitalized [10]. The use of high-dose loop diuretics to overcome diuretic resistance in treating ADHF can precipitate several side effects including hypokalemia, metabolic alkalosis, hypovolemia, hypotension, and worsening renal function [11].

Vasopressin receptor antagonists are a new class of drugs that produce selective water diuresis without affecting sodium and potassium excretion [12]. These drugs produce a net loss of free water that helps correct hyponatremia [13,14]. This class of agents can aid the treatment of hyponatremia in hospitalized ADHF patients. Currently, Vasopressin receptor antagonists are not approved for the treatment of hyponatremia secondary to heart failure unless hyponatremia is symptomatic or serum sodium reaches below 120 mEq/L. This agent is also not yet approved for the treatment of ADHF. The European Society of Cardiology and the American College of Cardiology does not endorse the use of this agent in the acute management of heart failure. The use of this agent in ADHF treatment is only mentioned in the 2017 report of the Japan Heart Failure Society Guideline (Class of Recommendation II a, Level of Evidence C), in which one study reported the use of this agent in ADHF. Tolvaptan, a vasopressin receptor antagonist used in this study has been reported to reduce the dose of furosemide in ADHF patients with CKD stage 3 with an eGFR of 15 to 60 mL/min [15,16]. Currently, there is no data regarding the effect of tolvaptan in ADHF patients in Indonesia.

In this study, we aim to further explore the role of tolvaptan, in the treatment of hyponatremia secondary to acutely decompensated heart failure.

## Methods

This study is a retrospective cohort study that included all patients with acute decompensated heart failure with hyponatremia who were hospitalized at National Cardiovascular Center Harapan Kita ( NCCHK) between January 2014 to May 2017. This study was approved by NCCHK Research Ethics Committee, ethical approval letter no. LB.02.01/VII/220/KEP.063/2017. Medical records were used to collect patients' characteristics, clinical parameters, echocardiographic parameters, and laboratory results. Study samples were obtained using consecutive sampling methods retrospectively from the medical records. The inclusion criteria were (1) adult patients (2) patients admitted to NCCHK Emergency Department with the diagnosis of acute decompensated heart failure (3) patients with serum sodium < 135 mEq/L (4) patients given tolvaptan. The exclusion criteria were (1) patients with serum creatinine exceeding 3.5 mg/dl or undergoing renal replacement therapy (2) patients with sepsis (3) patients with chronic obstructive pulmonary disease.

Acute decompensated heart failure in this study was defined as a progressive worsening of chronic heart failure that has been medically treated with evidence of systemic or pulmonary congestion. Hyponatremia in this study was defined as a serum sodium level under 135 mmol/L. A total of 71 patients were given tolvaptan. The agent of intervention in this study is the administration of tolvaptan, a competitive vasopressin receptor 2 (V2) antagonist (SAMSCA TM, Otsuka Pharmaceuticals, Japan). the administration was performed in the Adult Ward of National Cardiovascular Center Harapan Kita, decision and time to Administer this drug were determined by the Clinical Cardiology team.

Tolvaptan administration was based on the ability to afford this agent for the study subjects. Patients were offered tolvaptan if diuresis were not responsive to IV furosemide (Diuretic Resistance), renal dose dopamine, and thiazide on the third day of admission to the ward; patients who were able to afford tolvaptan were given tolvaptan. Diuretic resistance in this study is defined as a urinary diuretic response of less than 1400 ml per 40 mg of furosemide or equivalent [^17^]. Tolvaptan was administered in 15 mg dose tablets once daily to patients in this study until the point of optimal clinical decongestion as evaluated by the treating physicians. In the cases of patients initially treated in the intensive care unit, tolvaptan administration was initiated after the patient had been transferred from the ICU to the ward. The delay of tolvaptan initiation until the patient is in the ward is caused because our study team consists only of a clinical cardiologist who manages patients in the wards, while intensive care units are managed by cardiologists specializing in intensive care who is not part of our study team.

We performed analysis on the onset of tolvaptan administration in our study subjects. Subjects were then dichotomized into early vs late administration groups based on the median of onset of administration (5 th day). Comparison and analysis of baseline data and outcome of interests were then performed based on this dichotomy. Outcomes of interest in this study were (1) length of stay (2) nett sodium increase post tolvaptan administration (3)Death. These variables were then analyzed further using linear regression and multivariate analysis.

### Data analysis and statistics

In this study, we present numerical data with normal distribution in mean with standard deviation. Abnormally distributed numerical data were presented in the median with (minimum–maximum range). Categorical data are presented with total numbers and percentages. Several numerical variables were converted into categorical data using a cut-off value. Comparison of data between treatment and control groups was done using independent sample T-Test and Mann–Whitney Test for normal and abnormally distributed datasets, respectively. The effect on length of stay was analyzed using the Chi-square test with variables with P value < 0.25 or investigators' discretion included in multivariate analysis using linear regression. Data were analyzed using SPSS™ version 25 (IBM Corporations).

## Results

A total of 71 patients with hyponatremia secondary to ADHF were included in this study, and all patients were given tolvaptan. 37 patients were administered tolvaptan early (up until the fifth day of admission). 34 patients received tolvaptan after the fifth day of admission mean administration was 6.86th day and median administration was 5th day (Table 1). The mean left ventricular ejection fraction (LVEF) was 37.43 ± 21.11 vs 28.26 ± 15.21% in the early vs late administration group. Baseline serum creatinine was 1.79 + 0.69 vs 1.76 + 0.59 mg/dl p0.883 in the early vs late initiation group. From baseline characteristics of study subjects, no other significant differences in characteristics were observed (Table 2).

**Table 1:** Profile of onset of tolvaptan initiation on study subjects

Initiation of tolvaptan (day since admission to ward) n = 71	
Mean	6.86 th Day
Median	5 th day
Minimum	1 st day
Maximum	27 th day

**Table 2:** Baseline Characteristics

Variable	Early tolvaptan initiation	Late tolvaptan initiation	P value
	(n = 37)	N = 34	
Age, mean (SD), year	59.05 ± 13.61	51.82 ± 14.15	0.032
Males, n (%)	26 (70.3)	25 (73.5)	0.967
*Preexisting conditions, n (%)*			
Hypertension	20 (54.1)	18 (52.9)	1.000
Diabetes mellitus	19 (51.4)	17 (50)	1.000
Renal Insufficiency	18 (48.6)	22 (64.7)	0.232
Atrial Fibrillation	14 (37.8)	8 (23.5)	0.211
Baseline Serum Creatinine (mg/dl, mean ± SD)	(1.79 ± 0.69)	(1.76 ± 0.59)	0.883
*NYHA Class, n (%)*			
III	35 (94.6)	27 (79.4)	0.07
IV	2 (5.4)	7 (20.6)	0.07
*Cause of Heart Failure, n(%)*			
Ischemic Heart Disease	21 (56.8)	21 (61.8)	0.181
Non Ischemic Heart Disease	14 (37.8)	7 (20.6)	0.181
GUCH	2 1 (2.7)	1 (2.9)	0.181
Primary Cardiomyopathies	1 (2.7)	5 (14.7)	0.181
*Patient profile during admission*			
Systolic Blood Pressure at admission, median (min–max), mmHg	103.30 ± 21.51	101.47 ± 21.03	0.719
Left Ventricular Ejection Fraction at admission, mean ± SD (%)	37.43 ± 21.11	28.26 ± 15.21	0.039
Hemoglobin, mean (SD), g/dL	13.01 ± 2.26	12.07 ± 2.51	0.101
WBC on admission (count ± SD)	10,688 ± 4476	6400 ± 2990	0.156
Sodium during admission, mean ± SD (mEq/L)	126.24 ± 8.42	128.35 ± 5.74	0.219
*Medications during admission*			
Concomitant spironolactone administration	30 (81.1)	25 (73.5)	0.572
ACE Inhibitor	37 (100)	34 (100)	N/A
Beta Blocker	23 (62.2)	15 (14.1)	0.199
Admission to ICU / HCU during hospitalization	12 (32.4)	15 (44.1)	0.442

Although not statistically significant, patients with early tolvaptan administration showed trends of lower cumulative furosemide dose, average daily furosemide dose, and lower cumulative spironolactone dose. There were 3 deaths observed during the observation period, all of them belonging to the late initiation group. **(**Table 3**).**

**Table 3:** Comparison of outcome of interest between early (< 5 th day) and late (> 5th day) of tolvaptan initiation

	Early Initiation (n = 37)	Late initiation (n = 34)	P value
Length of stay (mean ± SD) (days)	8.86 ± 5.06	18.5 ± 9.05	0.001
Increase of Sodium Level, (mean ± SD), mEq/L	6.46 ± 6.69	3.68 ± 4.70	0.048
Cumulative Furosemide Dose (Mean ± SD), mg	1187.57 ± 1576.36	1698.38 ± 2111.43	0.256
Average Daily Furosemide Dose (Mean ± SD), mg	110.03 ± 90.47	86.84 ± 79.93	0.258
Cumulative spironolactone dose (mean ± SD), mg	31.67 ± 18.78	60.00 ± 100.13	0.175
Death	0(0)	3(8.8)	0.105

Multivariate linear regression analysis showed a statistically significant association between cumulative furosemide dose (mg), age, day of tolvaptan initiation, delta sodium since admission, and baseline Na Levels. This multivariate model showed good representativeness to study subjects with an R-value of 0.922, R [2] = 0.850, and ANOVA p value of < 0.001. Based on the current multivariate linear regression model, regarding the relationship between the onset of tolvaptan administration, each day of delay in tolvaptan administration will result in an increase in 0.946 days of length of stay (Table 4).

**Table 4:** The final model of multivariate analysis on length of stay

	Unstandardized coefficients		Standardized coefficient		
Variables	B	SE	B	t	P value
Cumulative Furosemide Dose (mg)	0.002	0.000	0.385	7.310	< 0.0001
Age (years)	–0.075	0.003	–0.124	–2.273	0.026
Day of tolvaptan initiation	0.946	0.073	0.680	12.888	< 0.0001
Delta Sodium since admission	–0.193	0.081	–0.132	–2.383	0.02
Baseline Na Levels	–0.229	0.067	–0.192	–3.349	0.001
R = 0.922 R [2] = 0.850 pANOVA = < 0.0001					

## Discussion

The main findings of the study are (1) patients who received early administration of tolvaptan have a lower length of stay (2) patients with early tolvaptan administration received a higher net increase in sodium during hospital stay (3) patients with early tolvaptan administration received a lower total dose of furosemide during the hospital stay. (4) delay of tolvaptan initiation will cause a higher length of stay.

We observed three deaths in the late tolvaptan initiation group, however, considering the relative proportion of death to the number of study subjects, we did not perform further analysis on this outcome.

The result of our bivariate and multivariate analysis showed that patients receiving early tolvaptan administration have a lower length of stay (8.86 ± 5.06 vs 18.5 ± 9.05 p0.001, respectively), and with each day of delay in tolvaptan administration since admission causing an increase in 0.946 days of stay in hospital. This benefit of LOS reduction is believed to be mediated by the aquaretic decongestion effect of AVP. Early resolution of decongestion will lead to earlier mobilization which in turn will lead to decreased LOS, a finding that is similar to the EVEREST, SALT-1, and SALT-2 studies [14,18,19].

Our analysis regarding serum sodium showed a higher increase in serum sodium in patients with early tolvaptan administration compared to late administration 6.46 ± 6.69 vs 3.68 ± 4.70 mEq/L p0.048, respectively. This shows that tolvaptan has a benefit in further increasing free water diuresis in patients with ADHF and also increasing serum sodium while not compromising electrolyte homeostasis as is usually the phenomenon with loop diuretics, early usage of tolvaptan is also associated with a trend of lower cumulative dose of furosemide in our study 1187.57 ± 1576.36vs 1698.38 ± 2111.43 mg p = 0.256 for early vs late tolvaptan administration, respectively. The use of this agent helps further augment the decongestive effort made by clinicians in the hospital as some patients with ADHF fail to achieve optimal clinical decongestion at the end of their hospital stay. This effect of decongestion augmentation using tolvaptan and the fact that fewer patients treated with this agent have hyponatremia during the end of their hospital stay is one of the main benefits of this agent highlighted by the 3 T Trial and the AQUA HF study [5,20].

Diuretic resistance is prevalent in ADHF patients, with studies reporting rates as high as 20–30%, it remains a challenge in the management of ADHF and is associated with a higher risk of subsequent death, rehospitalization, or renal complications secondary to heart failure. In overcoming this diuretic resistance, thiazide diuretic can be added to the loop diuretic regimen, a sequential nephron blockade, to curb renal adaptation of increased sodium reabsorption [21].

Diuretic resistance in ADHF occurs in the form of hypervolemic hyponatremia, in this state, low renal plasma flow and the subsequent RAAS activation with secondary renal sodium conservation are observed. This phenomenon, alongside multiple neurohormonal alterations secondary to the reduced renal plasma flow, caused increased activation of the sympathetic nervous system and the renin–angiotensin–aldosterone system, causing even further sodium and fluid retention with the subsequent congestive state with peripheral edema [1].

The occurrence of this phenomenon so-called cardiorenal syndrome is further aggravated in the face of ADHF, which is known as cardiorenal syndrome type 1. In this type, acute kidney injury occurs secondary to an increase in central venous pressure and increased interstitial pressure with subsequent tubular collapse and progressive decline in GFR [22–24].

The alteration of kidney function secondary to heart failure caused a dulled response of loop diuretics in patients with ADHF. The work of loop diuretics is impeded by the intense sodium and fluid reabsorption in the loop of Henle mediated by the RAAS and SNS secondary to low cardiac output states. 3 This activation of RAAS and SNS will also lead to increased release of AVP which will result in enhanced free water retention [25]. Tolvaptan is an arginine vasopressin antagonist with a selective property to the V2 receptor. These receptors are found on the renal collecting ducts and cause free water reabsorption leading to increased water retention [26].

The use of tolvaptan will enable clinicians to circumvent diuretic resistance by targeting sites of action in the kidneys that are independent of the loop of Henle by antagonism of the arginine vasopressin V2 receptor which promotes excretion of free water.

Tolvaptan is beneficial in curbing this “diuretic resistance” and reducing the length of hospitalizations in these patients. It is also interesting to note that one study found a stable response to tolvaptan administration regardless of the serum albumin level, in contrast, commonly used diuretics are highly protein-bound [27]. This finding is significant due to hypoalbuminemia being a common finding in heart failure patients. This notion is further supported by results of EVEREST, SALT-1, and SALT-2 studies which emphasize that (1) The use of tolvaptan was associated with a greater increase in sodium during treatment (2) use of tolvaptan was associated with a less stringent fluid restriction regimen during hospitalization (3) use of tolvaptan was associated with lower length of stay and (4) adverse events between treatment and control group were unequivocal [14,18].

A retrospective cohort study of the Kyoto Congestive Heart Failure (KCHF) registry, showed a non-statistically significant association between tolvaptan use and outcomes of all-cause death, cardiovascular death, and non-cardiovascular death at 90-day follow-up from hospitalization (adjusted HR = 1.53, 95% CI = 0.77–3.02, P = 0.22; adjusted HR = 2.00, 95% CI = 0.85–4.72, P = 0.11; adjusted HR = 0.82, 95% CI = 0.24–2.74, P = 0.74) [28]. However, these results must be interpreted with caution considering several factors. The KCHF study did not have a clear assignment protocol of tolvaptan to study subjects, furthermore, the tolvaptan/intervention arm of the study consisted both of early (< 48 h) and late (> 48 h) administration of tolvaptan. The inclusion of late tolvaptan usage in the intervention group has the potential to equivocate the actual effect of tolvaptan due to less benefit in late tolvaptan usage in patients compared to early, a similar phenomenon can also be observed in this study. Also, subjects given tolvaptan in this study were older and higher in BNP and NT pro-BNP levels. The intervention group in this study also consisted of more patients with valvular heart disease, diabetes, chronic kidney disease, congestive symptoms, and higher NYHA functional class. Compared to our study, more patients suffer from valvular heart disease across both study groups in this study with moderate-severe mitral regurgitation reaching 31.9% and 40.4% and moderate-severe regurgitation reaching 24% and 35% in the control and treatment groups, respectively [28]. The result of this study shows a reduction in length of stay in the early use of tolvaptan in ADHF patients, a result that is different from a recent study on the effect of door-to-tolvaptan time on short-term clinical outcome in patients with acute heart failure by Nomura et al. [29]. In their study, subjects were dichotomized based on early vs late tolvaptan administration with early defined as being under 6 h. A study by Nomura et al. fails to show any significant effect of tolvaptan use earlier than 6 h on total urine volume, length of stay, or worsening renal function. With the result of our study, the distinction between early and late tolvaptan usage would likely be in a matter of days, not hours, since in our comparison administration before the fifth day can still be beneficial to patients, in our opinion, likely, the equivocal nature of the result of a study by Nomura et al. stems from the fact that both comparison groups falls to an early timeline of tolvaptan administration.

In our study, the benefit of tolvaptan early initiation was observed as a reduction in length of stay in both bivariate (8.86 + 5.06 days vs 18.5 + 9.05 days, p < 0.001 for early and late initiation, respectively) and linear regression multivariate analysis (B -0.124, p0.026; R-value of 0.922, R2 = 0.850 and ANOVA p Value of < 0.001). Even with disparity in our study baseline characteristics of study subjects, with subjects in the intervention group having lower LVEF. This further solidifies the benefit of tolvaptan usage in ADHF patients across various ejection fraction profiles and degrees of heart failure. With this result, we concluded that disparity in our baseline characteristics of study subjects did not affect the results and interpretation of this study. However, a larger study with a randomized design on this subject will be needed in the future.

Based on the results of this study, it is beneficial to initiate tolvaptan in the ADHF regimen of therapy as early as possible, double-blind placebo randomized controlled trials should be done in the future to explore further the potential of tolvaptan in treating ADHF-related hyponatremia. Considering the promising result of tolvaptan in this study, we believe that in the future the use of tolvaptan in hyponatremic ADHF patients should be further explored in larger randomized controlled trials.

### Study limitation

The authors would like to acknowledge the limitations of this study; there was no matching of baseline clinical characteristics of study subjects done in this study. The administration of tolvaptan in this study is based on the ability to afford this agent by the patients, which caused a considerable lapse between study enrollment and analysis due to not many patients being able to afford this drug. Tolvaptan is not currently covered in our National Health Insurance coverage, since our hospital is a public hospital, most patients utilize the national health insurance for payment. Since the tolvaptan administration was based on an out-of-pocket payment, delays in administration also occur because some patients require time before deciding to pay for this drug after being offered. We would like to caution the readers in interpreting the results of this study based on the possibility of selection and channeling biases generated by the limitations of this study.

## Conclusions

Early administration of tolvaptan in treating hyponatremia in acutely decompensated heart failure patients is associated with a lower length of hospitalization and a higher increase in serum sodium of patients in hyponatremic ADHF patients.

## Data Availability

The data supporting the findings of this study are available from the corresponding author upon reasonable request from the Editor in Chief of the Journal.

## Abbreviations

ADHF: Acute decompensated heart failure
AUC: Area under the curve
AVP: Arginine vasopressin
BNP: N-terminal pro brain natriuretic peptide
NCCHK: National cardiovascular center harapan kita
NT PRO BNP: N-terminal pro brain natriuretic peptide
CVCU: Cardiovascular intensive care unit
HCU: High care unit
ICU: Intensive care unit
IWM: Intermediate ward (high care unit)
LOS: Length of stay
NYHA: New york heart association
RAAS: Renin–angiotensin–aldosterone system
ROC: Receiver operating curve
USD: United states dollars
